# Chemometrics: An Excavator in Temperature-Dependent Near-Infrared Spectroscopy

**DOI:** 10.3390/molecules27020452

**Published:** 2022-01-11

**Authors:** Yan Sun, Wensheng Cai, Xueguang Shao

**Affiliations:** Research Center for Analytical Sciences, Frontiers Science Center for New Organic Matter, College of Chemistry, Nankai University, Tianjin Key Laboratory of Biosensing and Molecular Recognition, State Key Laboratory of Medicinal Chemical Biology, Tianjin 300071, China; sunyan@mail.nankai.edu.cn (Y.S.); wscai@nankai.edu.cn (W.C.)

**Keywords:** temperature-dependent near-infrared spectroscopy, chemometrics, water structure, structural analysis, quantitative analysis

## Abstract

Temperature-dependent near-infrared (NIR) spectroscopy has been developed and taken as a powerful technique for analyzing the structure of water and the interactions in aqueous systems. Due to the overlapping of the peaks in NIR spectra, it is difficult to obtain the spectral features showing the structures and interactions. Chemometrics, therefore, is adopted to improve the spectral resolution and extract spectral information from the temperature-dependent NIR spectra for structural and quantitative analysis. In this review, works on chemometric studies for analyzing temperature-dependent NIR spectra were summarized. The temperature-induced spectral features of water structures can be extracted from the spectra with the help of chemometrics. Using the spectral variation of water with the temperature, the structural changes of small molecules, proteins, thermo-responsive polymers, and their interactions with water in aqueous solutions can be demonstrated. Furthermore, quantitative models between the spectra and the temperature or concentration can be established using the spectral variations of water and applied to determine the compositions in aqueous mixtures.

## 1. Introduction

Near-infrared (NIR) spectroscopy has been recognized as a powerful technique to study the structure of water [1]. Due to the strong absorption, the spectral information of the water structures with different hydrogen bonds can be measured, and the structures can be distinguished by analyzing the spectrum [1,2]. Aquaphotomics proposed by Tsenkova provides a framework for understanding the structural change of water caused by various perturbations such as temperature and solutes in aqueous and biological systems [3,4]. The spectral change of water can be used not only as a mirror to reflect the properties of the solutes in aqueous systems, but also as a probe to diagnose diseases or abnormalities non-invasively [5,6,7]. The spectral pattern describing the structures of water is used to measure the quality of water under different filtration treatments [8], to monitor the lactic acid bacteria fermentation in yogurt production [9], and to understand the physiological strategies of the resurrection plant during desiccation and rehydration procedure [10].

Temperature-dependent NIR spectroscopy is developed based on the temperature effect on the spectrum [11,12,13]. In 2010, the temperature dependency of NIR spectra on the temperature was studied, and a quantitative spectra–temperature relationship (QSTR) model between the NIR spectra of water and the temperature was established using partial least squares (PLS) regression [11,12]. Then, methods for analyzing the high-dimensional data matrices, including N-way principal component analysis (NPCA), parallel factor analysis (PARAFAC), and alternating trilinear decomposition (ATLD) were employed to analyze high-dimensional temperature-dependent NIR spectra [14]. The spectral variations induced by both the temperature and the concentration were obtained. The former can be used for structural analysis, and the latter can be used for building a calibration model for quantitative analysis. To simplify the calculation, low-dimensional algorithms were also proposed for analyzing high-dimensional temperature-dependent NIR spectral data. Mutual factor analysis (MFA) was proposed, and multilevel simultaneous component analysis (MSCA) was applied for the quantitative analysis of glucose solutions and serum samples [15,16,17]. A quantitative model was established for predicting the glucose content in aqueous and serum samples. Furthermore, the spectral variation of water induced by the temperature reflects the structural changes and interactions of water and solutes in aqueous solutions. Chemometric methods to improve the spectral resolution and extract temperature-induced spectral variations were studied for structural analysis, including continuous wavelet transform (CWT), independent component analysis (ICA), and Gaussian fitting.

In 2019, a review was published, in which chemometric methods and the applications in the resolution, quantitative, and structural analysis of temperature-dependent NIR spectra were summarized [13]. In this review, further works on extracting structural and quantitative information from temperature-dependent NIR spectra by chemometric methods were summarized. In temperature-dependent NIR spectroscopy, the perturbation of the temperature is taken as a source of spectral information reflecting the variation of systems. When the composition or concentration in the system changes, the temperature dependency of the spectra changes accordingly. Therefore, the temperature-induced spectral features can be used to reflect the properties of the systems. To capture the temperature-induced spectral information of water, chemometric algorithms were proposed for improving the resolution of NIR spectra. The spectral features of water reflecting the structural changes and interactions were obtained. Through the temperature-induced spectral variation, the interactions of water with small molecules, proteins, and polymers were investigated, and the role of water in the chemical and biological processes was revealed. Furthermore, quantitative determination can also be achieved using the spectral variation of water with the temperature or concentration and successfully applied to the quantification of the compositions in aqueous mixtures.

## 2. Structural Information of Water

The structure of water has been an interesting subject in chemistry and biology for decades due to the complex and flexible patterns of hydrogen bonding [18,19,20,21]. The effect of the temperature on the NIR spectrum of liquid water was studied as early as in 1925 [22], and over the past few decades, a number of works reported the temperature dependency of the NIR spectra of water [23,24,25,26,27,28]. Most of the researches focused on the absorption band around 6900 cm^−1^ measured at different temperatures, where an isosbestic point can be observed, implying the variation of the overlapped spectral components with the change of the temperature. With the help of chemometric methods, the spectral features of water structures with different hydrogen bonds were obtained, and the relative abundances of different water structures that change with the temperature were found [27,28]. Thus, the temperature-dependent NIR spectroscopy combined with chemometrics provides an efficient way to explore the structure of water in aqueous systems.

**Figure 1:**
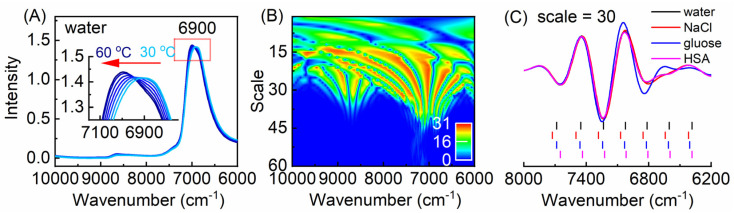
Temperature-independent near-infrared (NIR) spectra of water in the range of 6000–10,000 cm^−1^ measured from 30 to 60 °C (**A**), the fountain graph (**B**), and the selected variables for the samples of water, NaCl (5.8 g L^−1^), glucose (20 g L^−1^), and HSA (5.0 g L^−1^) solutions (**C**).

To investigate the effect of the temperature on the NIR spectra of water, a method for selecting the temperature-dependent variables from the temperature-dependent NIR spectra was developed. Figure 1A shows the temperature-dependent NIR spectra of water measured from 30 to 60 °C in the spectral range of 6000–10,000 cm^−1^. It can be seen that with the increase of the temperature, a shift of the peak around 6900 cm^−1^ to a higher wavenumber was observed, which is caused by the change of the overlapped spectral components corresponding to different water structures. To obtain the temperature-dependent information from the spectra, a method combined CWT and Monte-Carlo uninformative variable elimination (MC-UVE) was proposed for the selection of the temperature-dependent variables (wavenumbers) from the NIR spectra measured at different temperatures [29]. CWT was used to decompose the spectra into the spectral components with different frequencies, and then MC-UVE was employed to evaluate the importance of the variables in the quantitative model of the spectra and temperature. Figure 1B shows the stability of the transformed water spectra obtained by MC-UVE, which is named as “fountain graph”. In the fountain for the peak around 6900 cm^−1^, seven variables with a significant temperature dependency can be found. This indicates the complexity of water structures and suggests that there are different water species of which the spectral features change differently with the temperature. Furthermore, the temperature-dependent NIR spectra of the aqueous solutions containing NaCl, glucose, and human serum albumin (HSA) were investigated. Figure 1C shows the transformed spectra of water and solutions by CWT and the locations of the selected variables. It can be seen that the selected variables are located at similar but not identical wavenumbers for different solutions, indicating that the variables can be used for the discrimination of different solutions. Furthermore, using the selected variables, quantification can also be achieved. The results indicate that temperature-dependent NIR spectra can be severed as a mirror to reflect the complexity of water structures and identify the aqueous solutions of different compositions.

**Figure 2:**
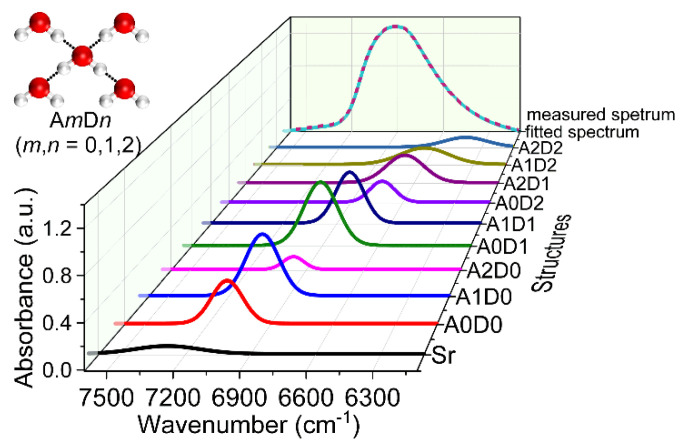
Result of Gaussian fitting for the NIR spectra of water measured at 30 °C.

To understand water structures in liquid water and aqueous solutions, Gaussian fitting was adopted to analyze the temperature-dependent NIR spectra of water. Six spectral components were used to fitting the spectra, corresponding to different water species with no (S_0_), one (S_1_), two (S_2_), three (S_3_), and four (S_4_) hydrogen bonds, as well as the rotation vibration (S_r_) of the water molecule [30]. To describe the complex water structures more exactly, a model was proposed by denoting the proton acceptor (oxygen) with A and the proton donor (hydrogen) with D. The water molecule with *m* hydrogen bonds on oxygen atom and *n* hydrogen bonds on hydrogen atom is represented by A*m*D*n*, where *m* and *n* equal to 0, 1, or 2. Therefore, nine water structures can be defined, i.e., A0D0, A0D1, A1D0, A0D2, A1D1, A2D0, A1D2, A2D1, and A2D2. Ten spectral components corresponding to the nine water structures and S_r_ were obtained from the temperature-dependent spectra of water and glucose solutions by Gaussian fitting with a knowledge-based genetic algorithm [31]. Figure 2 is one of the results obtained by the fitting. It can be seen that the fitted spectrum coincides well with the measured one. The integral intensity of the 10 peaks was investigated using the NIR spectra measured at different temperatures. With the increase of the temperature, the content of A0D0 increases, while that of A2D2 decreases, indicating the weakening of the hydrogen bonds and the dissociation of the water structures with more hydrogen bonds into that with less hydrogen bonds. Furthermore, through the variation of the spectral components of these water structures with the glucose concentration, the enhancement of the ordered (tetrahedral) hydrogen-bonded water structures induced by the interaction of water and glucose were found, providing a proof for the explanation of the protective effect of glucose on the bio-molecules in aqueous solutions.

CWT has been proven to be a powerful tool for the resolution enhancement in the spectral analysis [32,33,34,35]. To analyze the spectral features of water in mixtures of water and ethanol, the fourth-order derivative of the temperature-dependent NIR spectra of the mixtures was calculated by CWT [36]. The overlapped peaks were separated, and the spectral features of OH and CH with various intermolecular interactions were identified in the fourth derivative spectra. By fitting the derivative spectra of the mixtures by those of pure water and ethanol, the obtained coefficients for ethanol show a linear relation with the content, but those for water exhibit a non-linear relation, which provides clear evidence for the interactions of ethanol and water in the mixtures. Furthermore, from the residual spectra after the fitting, the structures of water species, aggregations of ethanol, hetero clusters of ethanol–water, and their variation with the content were analyzed. The residual spectra calculated by high-order derivatives provide a very good way to uncover the spectral information about the interactions. These results indicate that the spectral information of water structures with different hydrogen bonds can be extracted from the temperature-dependent NIR spectra, and the temperature-induced spectral features can be a probe to reveal the structural changes and interactions in aqueous solutions.

## 3. Interaction of Water and Solutes

Water plays an important role in chemical and biological processes. The interaction of water and solutes is of great significance for understanding the properties of aqueous solutions or bio-systems [37]. Due to the sensitivity of NIR spectra to water structures, the spectral changes of water under different perturbations can be a probe to reveal the interactions in aqueous solutions. The interaction of water and bio-organisms including carbohydrate molecules and oligopeptide was investigated using the NIR spectra of aqueous solutions measured at different concentrations and temperatures [30,31,38,39,40,41]. The spectral components related to different water structures were obtained from the NIR spectra. Through the variation of these structures with the temperature and the solute concentration, an increase of the tetrahedrally hydrogen-bonded water structure induced by bio-molecules was observed, showing that the thermal stability of water structures may be enhanced in bio-systems.

The interaction of water and ethanol was studied using the temperature-dependent NIR spectroscopy with high-order chemometric algorithms, including NPCA, PARAFAC, and ATLD [14]. The spectral features of water and ethanol in the mixtures were obtained by the three methods. Through the variation of the spectra with the concentration, it was revealed that ethanol promotes the formation of water clusters. To obtain more spectral information of the interactions of water and ethanol, a new method was proposed based on the rotation of the loadings in principal component analysis (PCA) [42]. The calculated spectra were found to be more reliable to reflect the structures in the mixture, from which the spectral features of different water species (S_0_–S_4_), ethanol clusters, and the interaction of OH and CH groups were observed. Through the difference between the calculated and experimental spectra, it was found that, when ethanol is added into water, the contents of large water clusters with two, three, and four hydrogen bonds increase, and the interaction of water and ethanol varies nonlinearly with the concentration.

The structure of water at low temperatures is related to many special phenomena. For example, the freezing point of water reduces when an antifreeze is added, and water in polar fish does not freeze below the freezing point. The structure of water at low temperatures and the mechanism of the cryoprotectant dimethyl sulfoxide (DMSO) in reducing the freezing point of water were investigated using the NIR spectra measured at low temperatures [43]. CWT was adopted to enhance the resolution of the NIR spectra. The spectral features reflecting the interaction of DMSO and water were found from the resolution-enhanced spectra, and two hydrogen-bonded DMSO–water structures (DW2 and D2W) were identified in the mixtures with different DMSO/water ratios. Through the variation of the spectral features, it was found that DW2 structure inhibits the formation of tetrahedral water structures at low temperatures, which may be the reason for DMSO reducing the freezing point of the mixture. To further understand the effect of protein on the antifreezing performance of the DMSO–water system, the effect of formamide (FA) on the hydrogen bonding of DMSO and water was studied [44]. From the resolution-enhanced spectra by CWT, the spectral feature of the interaction of DMSO and water (S=O…H–O) was observed. When FA exists in the mixture, the intensity of the peak decreases with the increase of FA content, indicating that FA may replace the water molecules to form the hydrogen bond of S=O and H–N. Furthermore, the spectra of the three-component mixtures were analyzed by ATLD. Two varying spectral features due to water and DMSO were obtained, but the spectral feature variation with the content of FA was not found. This result implies that, although FA may reduce slightly the antifreezing effect, DMSO is still the key component to prevent water from icing.

## 4. Structural Change of Water in the Aggregation of Proteins and Polymers

Compared with that of small molecules and water, the interaction of macromolecule and water is more complex, and the interactions are easier to be affected by temperature. For understanding the phase transition mechanism and the role of water in the aggregation of proteins and polymers, the variation of water structures in the aggregation process was studied by using temperature-dependent NIR spectroscopy and chemometrics. The structural change of water in the thermal denaturation of proteins was investigated by analyzing the temperature-dependent NIR spectra of HSA and ovalbumin (OVA) solutions [45,46,47]. From the resolution-enhanced spectra by CWT, the spectral features of the protein (α-helix and β-sheet) and different water species (S_0_–S_4_) were observed. Through the variation of the peak intensities related to water species with temperatures, the structural change of the proteins was revealed, indicating that water can be a probe for investigating the structural variation of the proteins in aqueous solutions.

Tau is a class of intrinsically disordered proteins, of which the mistaken aggregation leads to neurodegenerative diseases, typically Alzheimer’s disease (AD). Investigating the interaction between tau protein and water during the aggregation is helpful to understand the pathogenesis of the disease. The variation of hydration water during the aggregation of the core fragment of tau, R2/wt, induced by heparin, was investigated using NIR spectroscopy combined with PCA and two-dimensional (2D) correlation spectroscopy [48]. The spectral information due to the structural change of R2/wt was observed from the resolution-enhanced NIR spectra, showing a two-stage variation during the aggregation. Furthermore, the spectral features of water species with one and two hydrogen bonds around NH and CH groups were found in the loadings of PCA, and the variation of the water species during the aggregation was analyzed by 2D correlation spectroscopy. Water species with one hydrogen bond change before the water molecules with free OH and with two hydrogen bonds. The result demonstrates that the hydrogen-bonded water with the NH groups disassociates at first, leading to the formation of the β-sheet structure, and then the hydration water around CH groups releases. This result shows the mechanism for the aggregation of tau protein, i.e., the dehydration of NH groups changes the hydrogen bonding network of the hydration water, and then, the water molecules near the hydrophobic side chains release from the R2/wt, resulting in the formation of the ordered amyloid fibers.

Biological processes such as protein folding mostly occur in cells. Water in a cell has different structures from that in bulk water because of the crowding and confined environment, of which the effect on the structure of biomolecules is considered to be the main reason for explaining the stability and activity of biomolecules [37]. To understand the function of water on the thermal stability of the proteins in a confined environment, the water structures in reverse micelles (RMs) were studied by temperature-dependent NIR spectroscopy [49]. The NIR spectra of aqueous solutions and RMs containing bovine serum albumin (BSA), HSA, and OVA were measured at different temperatures. After enhancing the resolution of the spectra, the spectral features of α-helix in proteins and water species with non-hydrogen bond (NHB), weak hydrogen bonds (WHB), and strong hydrogen bonds (SHB) were observed. The intensity change of the α-helix with the temperature shows a clear denaturation of the protein in aqueous solutions, but not in RMs, indicating the effect of the confined environment on the stability of the proteins. To further understand the water structures in RMs, PCA was performed on the transformed spectra. From the result, the spectral feature of a specific water structure in RMs was found, i.e., the bridging water connecting NH in the protein and S=O in the inner surface of RMs, which only exists in RMs with the protein. This water structure may be the reason for enhancing the thermal stability of the protein in RMs.

Temperature-sensitive polymers exhibit phase separation in aqueous solutions above the lower critical solution temperature (LCST). The interaction of water and the polymer is supposed to be the key factor in driving the aggregation. The temperature-dependent NIR spectra of poly(N,N-dimethylaminoethyl methacrylate) (PDMAEMA) in aqueous solutions were measured for investigating their interactions during the aggregation [50]. The spectral changes of the polymer and water during the aggregation with the temperature were analyzed by NPCA. In a low-concentration solution, a two-stage conformational change was observed in the phase transition, i.e., the hydrated chains tend to form an intermediate state of the loose hydrophobic structure and then aggregate into a micelle at the LCST. In the aggregation process, the S_2_ water species increases gradually and then a sudden decrease occurs after the LCST, indicating that S_2_ plays an important role in the formation of the intermediate. S_2_ water species acts as a bridge to connect the polymer chains in the loose hydrophobic structure, and the dissociation of the S_2_ at high temperatures leads to the formation of the micelle. Furthermore, the effect of urea on the interaction of water and the polymer poly(N-isopropyl acrylamide) (PNIPAM) was studied by NPCA for understanding the denaturation in aqueous environments [51]. The results indicate that the water species with three hydrogen bonds (S_3_) plays an important role in the stabilization of PNIPAM, which may connect the NH and CO groups in the polymer. The S_3_ water species stabilizes the coil state of the polymer, and the release of the species leads to the phase transition. When urea is added, urea may reduce the S_3_ content by the hydrogen bonding with the hydrophilic groups in the polymer, thus leading to a phase transition at a lower temperature.

## 5. Quantitative Analysis of Aqueous Solutions

NIR spectroscopy and chemomtric methods have been extensively used for quantitative determination in various fields [52,53], in which multivariate analysis is generally employed. In the analysis of aqueous solutions, the NIR spectrum contains the spectral information of structures and interactions. Both the information is related to the quantity of the composition. Therefore, quantitative determination can be achieved, with the aid of chemometrics, by analyzing the temperature-induced spectral changes in temperature-dependent NIR spectroscopy. A QSTR model between NIR spectra and the temperature was established based on PLS regression and applied to the quantitative determination of the compositions in aqueous solutions of methanol, ethanol, n-hexane, and their mixtures [11,12]. The results show that both the temperature and the quantity of the composition in mixture solutions can be predicted using the models. 

The temperature-dependent NIR spectra measured at different conditions are generally composed of high-dimensional data. For example, the spectral data of a group of samples with different concentrations measured at different temperatures are a three-way matrix with the dimension of wavenumber, concentration, and temperature. Thus, high-order chemometric algorithms have been employed to deal with high-dimensional data. NPCA, PARAFAC, and ATLD were adopted to explore the spectral information from the temperature-dependent NIR spectra of a binary water–ethanol and a ternary water–ethanol–isopropanol mixture solutions [14]. The temperature- and concentration-induced spectral variations were obtained by these algorithms, and the quantitative model was successfully built. For the four-way data array of the ternary mixtures, the algorithms were also proven to be a powerful tool for extracting the quantitative information from the spectral data. The result indicates that high-order chemometric algorithm may be a powerful tool for resolving the temperature-dependent NIR spectra to obtain the quantitative information of aqueous solutions.

In order to simplify the calculation, two-dimensional algorithms were also proposed for analyzing high-dimensional data. MFA was developed for the analysis of temperature-dependent NIR spectra [15,16]. The method unfolds a high-dimensional data array into a combined data matrix and then extracts the common spectral feature contained in the spectra of different temperatures or different concentrations by PCA. The relative quantity of the extracted spectral feature can be used to build the calibration model for quantitative analysis. The method of MFA was employed for the quantitative determination of both low- and high-concentration glucose solutions. The result shows that MFA can achieve the accurate quantification of the glucose content even in low-concentration solutions. Besides, the feasibility of the method was also validated using human serum samples. A calibration model with a good correlation coefficient was obtained for the measurement of the glucose content. More importantly, the calculations were based on the spectral information of water, demonstrating that MFA provides a potential way for detecting the components in complex bio-systems.

High-dimensional data can also be analyzed by MSCA through unfolding a data array into a two-dimensional matrix. A two-level MSCA model was employed to capture the temperature- and concentration-induced spectral variations in the spectra of water–ethanol–isopropanol mixtures [54]. The MSCA contains a between-temperature (QSTR) model describing the effect of the temperature and a within-temperature (QSCR) model describing the concentration variation. In the within-temperature model, the temperature-induced spectral changes are minimized, and the concentration-induced changes can be reflected. Therefore, quantitative analysis can be achieved by the coefficients in the model. The two-level MSCA was also used to detect glucose in aqueous glucose solutions and human serum samples [17]. Using the spectral changes of water, the relationship between the temperature or concentration and NIR spectra can be effectively described by the QSTR model from the first level and the QSCR model from the second level model.

**Figure 3:**
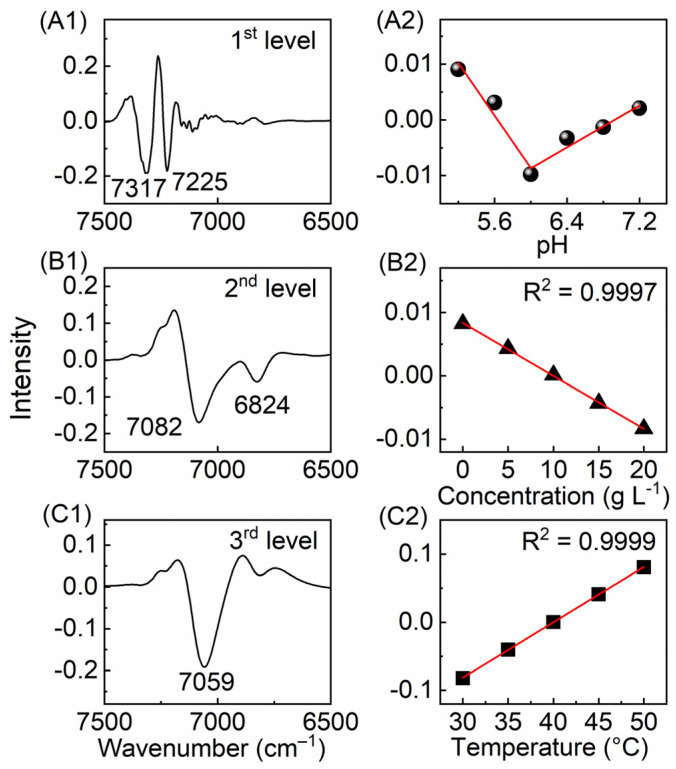
The loadings (**A1**–**C1**) and scores (**A2**–**C2**) of the three-level models obtained by three-level multilevel simultaneous component analysis (3-MSCA).

Furthermore, the NIR spectra changing with more factors were analyzed by MSCA. The NIR spectra of proline aqueous solutions in different pH and concentrations were measured at different temperatures [55]. A three-level MSCA (3-MSCA) model was established to investigate the pH-, concentration-, and temperature-induced spectral variations. Figure 3 shows the loadings and scores of the three-level models by the method. The first loading of the first-level model describes mainly the spectral information of the CH_2_ groups in proline. The corresponding score goes down first and then up at the isoelectric point, which is due to the structural changes of the proline. The second loading from the first-level model is mainly composed of the spectral information of water, and the corresponding score has the same inflection, which is related to the structural change of water with pH. The first-level model gives a good description of the effect of pH on the spectra, and the spectral changes can be used to analyze the structural change of proline and water. Figure 3(B1,B2) show the first loading and score of the second-level model. The former shows the spectral information of water, and the latter shows the variation of the spectral component with the concentration. The perfect quantitative relationship was obtained, which is a good proof that the interaction between water and proline can be used to describe the change of the composition quantitatively. After the pH- and concentration-induced spectral changes are minimized, the temperature-induced change of water is reflected in the loading and score of the third-level model, as shown in Figure 3(C1,C2), respectively. The relationship is a good QSTR model to quantitatively describe the change of the spectra with the temperature and to predict the temperature of the solution. Therefore, by extracting the information about the structural change of water with three factors in proline solutions, the quantitative models for the determination of pH, concentration, and temperature were obtained. The results further demonstrated that water can be a good probe for sensing quantitative information.

## 6. Conclusions

Temperature-dependent NIR spectroscopy provides a powerful tool for aquaphotomic studies in both the structural analysis and quantitative determination of aqueous systems. Chemometric methods can be a powerful excavator to mine the temperature-induced information from the overlapping NIR spectra. The spectral features of water structures with different hydrogen bonds can be captured. Through the spectral variation of the water, the structural changes and interactions of water and small molecules, proteins and thermo-responsive polymers can be obtained. Furthermore, quantitative determination can also be achieved using the spectral variation of water with the temperature, concentration, and pH. With the advance of chemometrics, temperature-dependent NIR spectroscopy may be a promising technique for quantitative determination and understanding the properties or functions of analytes in aqueous solutions and biological systems.

## Data Availability

Not applicable.
